# Open surgery for carpal tunnel syndrome: is it necessary to release the antebrachial fascia? A randomized clinical trial study

**DOI:** 10.3389/fsurg.2024.1300972

**Published:** 2024-11-27

**Authors:** Mehran Razavipour, Sadegh Taheri, Amirsaleh Abdollahi, Yazdan Yahaghi

**Affiliations:** ^1^ Department of Orthopedics, Orthopedic Research Center, Mazandaran University of Medical Sciences, Sari, Iran; ^2^ Department of Orthopedics, Mazandaran University of Medical Sciences, Sari, Iran; ^3^ Department of Orthopedics, Student Research Committee, School of Medicine, Mazandaran University of Medical Sciences, Sari, Iran

**Keywords:** carpal tunnel syndrome, open surgery, antebrachial fascia, outcome, small incision

## Abstract

**Background:**

Open surgery for carpal tunnel syndrome (CTS) has historically involved release of the antebrachial fascia. The benefit of antebrachial fascia release in CTS surgery is still controversial. So, this study was designed to evaluate this hypothesis.

**Methods:**

The study was designed as a two-arm randomized clinical trial study. Patients diagnosed with bilateral carpal tunnel syndrome were enrolled in the study, while those under 18 years of age and those with a history of carpal tunnel release, trauma to the spine, shoulder, or elbow, rheumatologic disease, inflammatory arthropathy, and CTS onset during pregnancy were excluded. The hands of the eligible patients were randomly assigned to two surgical groups. In the first group, the antebrachial fascia was opened to the proximal part, while in the second group, the fascia was opened from the central part of the deep layer to the distal volar part of the wrist. Pain severity, grip and pinch strength, symptom severity, and functional status were evaluated by the visual analog scale, the SAEHAN® hydraulic handgrip and pinch dynamometer, and the Boston Carpal Tunnel Questionnaire (BCTQ), respectively, at the baseline and 1, 3, and 6 months after surgery.

**Results:**

Finally, 230 patients (220 women and 10 men, 460 hands) completed the study. The mean age of the patients was 50.4 ± 8.4 years. In both open surgery groups with and without antebrachial fascia release, the grip and pinch strength, BCTQ scores, and pain severity significantly improved at the end of the study (*P* < 0.01), but there was no statistically significant difference between the two groups (*P* > 0.05). Patient satisfaction improved in both groups; again, no significant difference was observed between the two groups (*P* > 0.05).

**Conclusion:**

Both open CTS surgery with and without antebrachial fascia release show the same clinical and functional outcomes. Therefore, avoiding the release of the antebrachial fascia preserves proprioception and prevents iatrogenic injury to the median nerve and its branches. Conversely, a blunt release of the antebrachial fascia does not adversely affect the outcome.

**Clinical Trial Registration:**

https://irct.behdasht.gov.ir/search/result?query=@irct_id:IRCT2012103111341N1, Identifier: IRCTID: IRCT2012103111341N1.

## Introduction

Carpal tunnel syndrome (CTS) is a symptomatic compression neuropathy of the median nerve in the wrist area, with a prevalence of 3.8% in the general population and an annual incidence of 276 per 10,000 person-years (1). CTS is more common in women than in men, with the peak age occurring between 40 and 60 years (2). It usually occurs bilaterally, affecting daily activities and reducing the quality of life of the patients (3, 4).

The main purpose of CTS treatment is to remove the pressure from the median nerve, which surgical procedures can achieve. Conservative treatments, including wrist splinting, local steroid injections, and oral treatment with non-steroidal anti-inflammatory drugs (NSAIDs), are recommended for mild to moderate CTS. Surgical treatments, such as open or endoscopic carpal tunnel release (CTR), have a higher success rate than conservative methods but may be associated with pain, weakness, infection, or nerve damage. Endoscopic surgery may result in a faster return to previous activities, but it has some limitations that need further study (5–7). Although some new alternatives to traditional surgical techniques have been developed to reduce complications, like ultrasound-guided surgery, these techniques are still under investigation (8).

Various studies have reported that open surgical treatments are more beneficial and effective than conservative treatments (9–11).

Carpal tunnel release surgery is based on reducing pressure on the median nerve through the release of fibrous structures. There are three layers of fibrous structures on the palmar side of the hands and forearms, which include the superficial layer of the fascia, the middle layer (palmar aponeurosis and antebrachial fascia), and the deep layer (12, 13). Studies have shown that the deep layer exerts a constrictive effect and should be cut to reduce the pressure on the nerve, while the middle layer plays a proprioceptive role. Therefore, damage to the middle layer may damage nerve structures and may contribute to increased morbidity (14, 15). Although incomplete release is the most common cause of ineffective surgical outcomes, the depth of release is not yet well known. Studies suggest that the removal of clinical pressure only occurs when the central and distal parts of the deep layers are released (16–19). Today, various surgical approaches are used for the treatment of CTS. Standard open surgery with a curvilinear incision remains the preferred surgical technique for many surgeons. However, it may be associated with several complications, including pain, scarring, tenderness, or patient dissatisfaction (20, 21).

Considering the advantages and disadvantages of carpal tunnel release, the preferred surgical procedure still needs to be well defined (22). This study aimed to compare the clinical outcomes and patient satisfaction in open carpal tunnel surgery by minimal access with the release of the antebrachial fascia vs. minimal access without the release of the antebrachial fascia.

## Methods

### Study design

The study was designed as a two-arm randomized clinical trial study. This study was conducted between 2017 and 2021. Patients diagnosed with bilateral carpal tunnel syndrome who were referred to the Orthopedic Clinic of Imam Khomeini Hospital of Sari, Iran, were enrolled in the study (according to the latest statistics, Sari has a population of 310,000, and our center serves as a referral point for all patients in the Mazandaran Province, which has a population of approximately 3 million). Inclusion criteria included moderate to severe bilateral CTS, confirmed by the electrophysiological studies, lack of response or recurrence after conservative treatment for at least 3 months, loss of sensation, or numbness at the site of the median nerve, along with positive Tinel's and Phalen's tests. Patients under 18 years of age and those with a history of carpal tunnel release, trauma to the spine, shoulder, or elbow, rheumatologic disease, inflammatory arthropathy, and CTS onset during pregnancy were excluded from the study.

The hands of the eligible patients were randomly assigned to two surgical groups using a simple randomization technique via the sealed envelope method. The operating room received the type of surgery in a sealed envelope, which contained the hand side (left or right) and the kind of CTS surgery (with or without the release of the antebrachial fascia). Before the operation, the sealed envelope was handed to the surgeon to determine which method would be performed. As described, each patient's hand was randomly assigned to either the case group (open surgery without the release of the antebrachial fascia) or the control group (open surgery with the release of the antebrachial fascia). Patients were encouraged to move their hands and fingers after the surgery.

### Surgical technique

**Figure 1:**
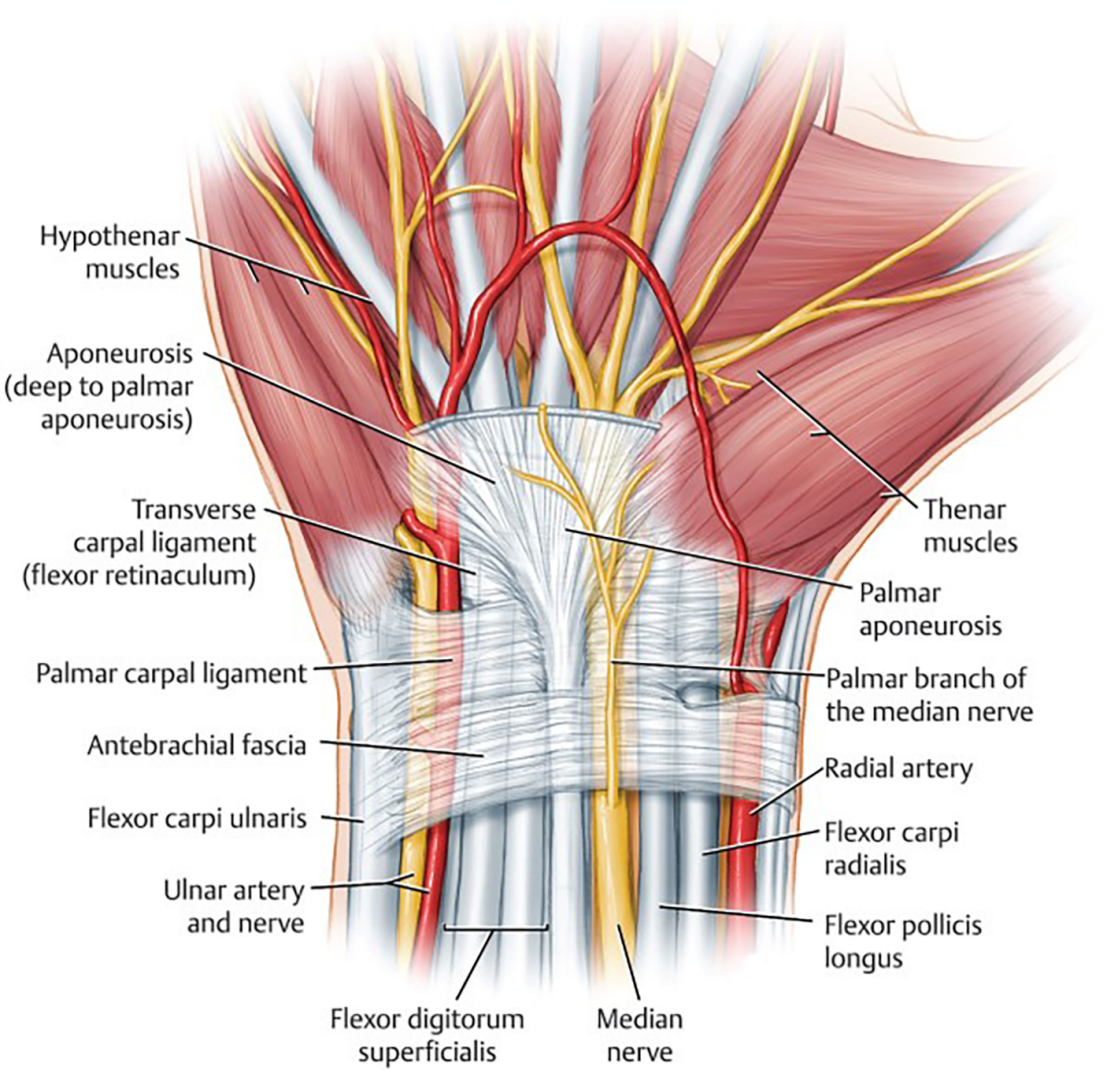
In the traditional approach to carpal tunnel release surgery, the surgical procedure involves the incision and division of the ligamentous structures, specifically the transverse carpal ligament and the antebrachial fascia, that exert pressure on the carpal tunnel. This surgical maneuver is performed to alleviate compression within the carpal tunnel, thereby creating additional space for the passage of the median nerve and its associated tendons. It is worth noting, however, that the incision and division of the antebrachial fascia are typically executed in a rather blunt manner, and this technique may carry the potential risk of causing nerve damage. Moreover, certain research findings suggest that the antebrachial fascia may not exert a compressive effect on the median nerve (14), thus raising questions about the necessity of incising and releasing this fascial structure during the procedure.

**Figure 2:**
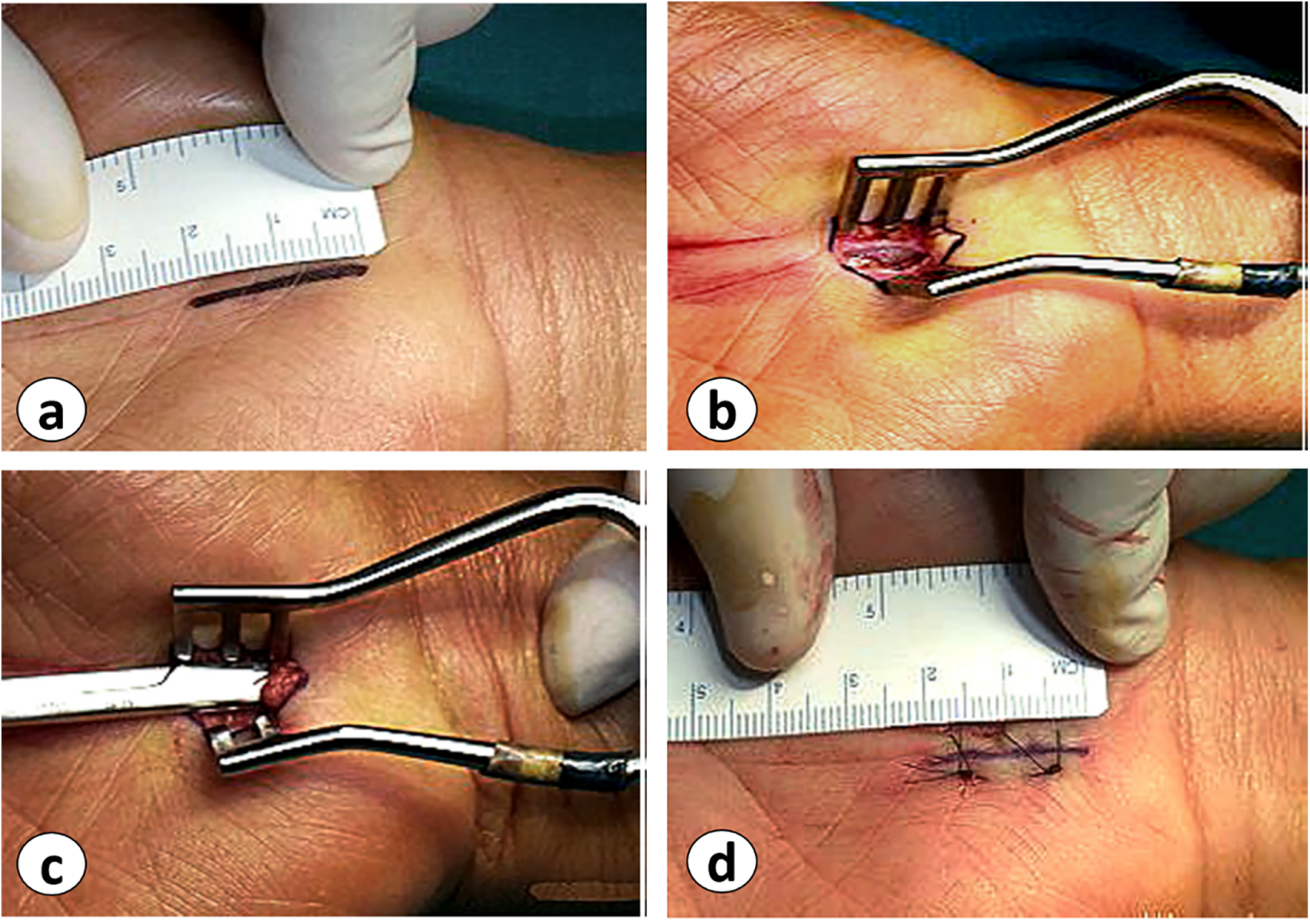
Description of the surgical technique: **(a)** CTR incision, **(b)** cutting of the subcutaneous tissue to find the palmar fascia and CTL, **(c)** median nerve observation after cutting the CTL, and **(d)** suturing after performing the CTR with or without releasing the antebrachial fascia.

Regarding the anatomy of the transverse palmar ligament and the antebrachial fascia (Figure 1), a 2-cm incision was made on the proximal palmar side of the hand over the transverse ligament. The incision started distal to the proximal edge of Kaplan's cardinal line. The line was drawn with the thumb abducted to the radial side, and then the line was traced at the radial margin of the fourth metacarpal bone (Figure 2a). After making the skin incision, the subcutaneous tissue was cut with a No. 15 razor, and two cutting edges were fixed with a retractor. The palmar fascia was opened, and the transverse carpal ligament (CTL) was detected (Figure 2b). The transverse carpal ligament was cut to access the carpal tunnel space, and then the median nerve was observed (Figure 2c). The transverse carpal ligament was opened to the distal part with scissors. In the first group, the fascia was bluntly opened proximally (antebrachial fascia) with scissors. In contrast, in the second group, only the distal and central portions were released, with the distal volar wrist crisis marking the margin of the antebrachial fascia. The incision site was sutured with 4/0 nylon yarn, and a bandage was applied (Figure 2d).

### Outcome assessment

Primary outcomes included pain severity, grip and pinch strength, symptom severity, and functional status. All of these items were assessed at the baseline and 1, 3, and 6 months after surgery. Pain severity was measured by the visual analog scale (VAS), a self-report questionnaire, which scored from 0 (no pain) to 10 (worst pain). Grip and pinch strength were measured in kilograms using the SAEHAN® hydraulic handgrip dynamometer (SAEHAN Corporation, Korea, Model SH5001) and SAEHAN® hydraulic pinch dynamometer (SAEHAN Corporation, Korea, Model SH5005). All dynamometers were new and calibrated according to the manufacturer’s instructions.

Symptom severity and functional status were evaluated using the Boston Carpal Tunnel Questionnaire (BCTQ) (23). The BCTQ is a self-administered tool consisting of 11 items for symptom severity scores (SSS) and 8 items for functional status scale (FSS) (24). Each item has five options, scaled between 1 (no problem) and 5 (worst condition). The mean scores are calculated for symptom severity and functional status. The higher the mean score, the greater the severity and disability of the patient (17, 18). We used the Persian version of the BCTQ, which has been validated previously, offering reasonable reliability, sensitivity, and internal consistency (19, 20, 25).

Secondary outcomes were patient satisfaction, bleeding, infection, and neurological or vascular complications. Post-surgical complications were checked at the time of discharge, in the presence of symptoms, and during follow-up visits. Patient satisfaction was evaluated using a numerical rating scale (NRS), a self-report questionnaire, which scored from 0 (dissatisfaction) to 10 (satisfaction). The satisfaction score was evaluated at 1, 3, and 6 months after surgery.

### Ethical considerations

The study protocol adhered to the Declaration of Helsinki (1989 revision) and was approved by the medical ethics committee of the Mazandaran University of Medical Sciences, Sari, Iran (reference number: CT-92-6709). The trial was registered in the Iranian Registry of Clinical Trials (registration ID: IRCT2012103111341N1). The treatment method and its complications were explained to the patients, and all of them signed written informed consent.

### Statistical analysis

Data analysis was performed using SPSS version 20 software. The normality of quantitative data was checked using the Kolmogorov–Smirnov test. Demographic and clinical characteristics were presented as mean ± standard deviation (SD) for continuous variables. Quantitative data were compared within and between the groups using general linear models, independent, and paired samples *t*-tests. *P*-values <0.05 were considered statistically significant.

## Results

In total, 250 patients were evaluated, and 245 met the inclusion criteria of the study. A total of 10 patients refused to participate, and five left during follow-up. Finally, 230 patients (220 women and 10 men, 460 hands) completed the study. The mean age of the patients was 50.47 ± 8.47 years (median 52 years). Since each patient's hand was randomly assigned to a group, the age, gender, and socioeconomic status were perfectly matched between groups.

### Primary outcomes

**Figure 3:**
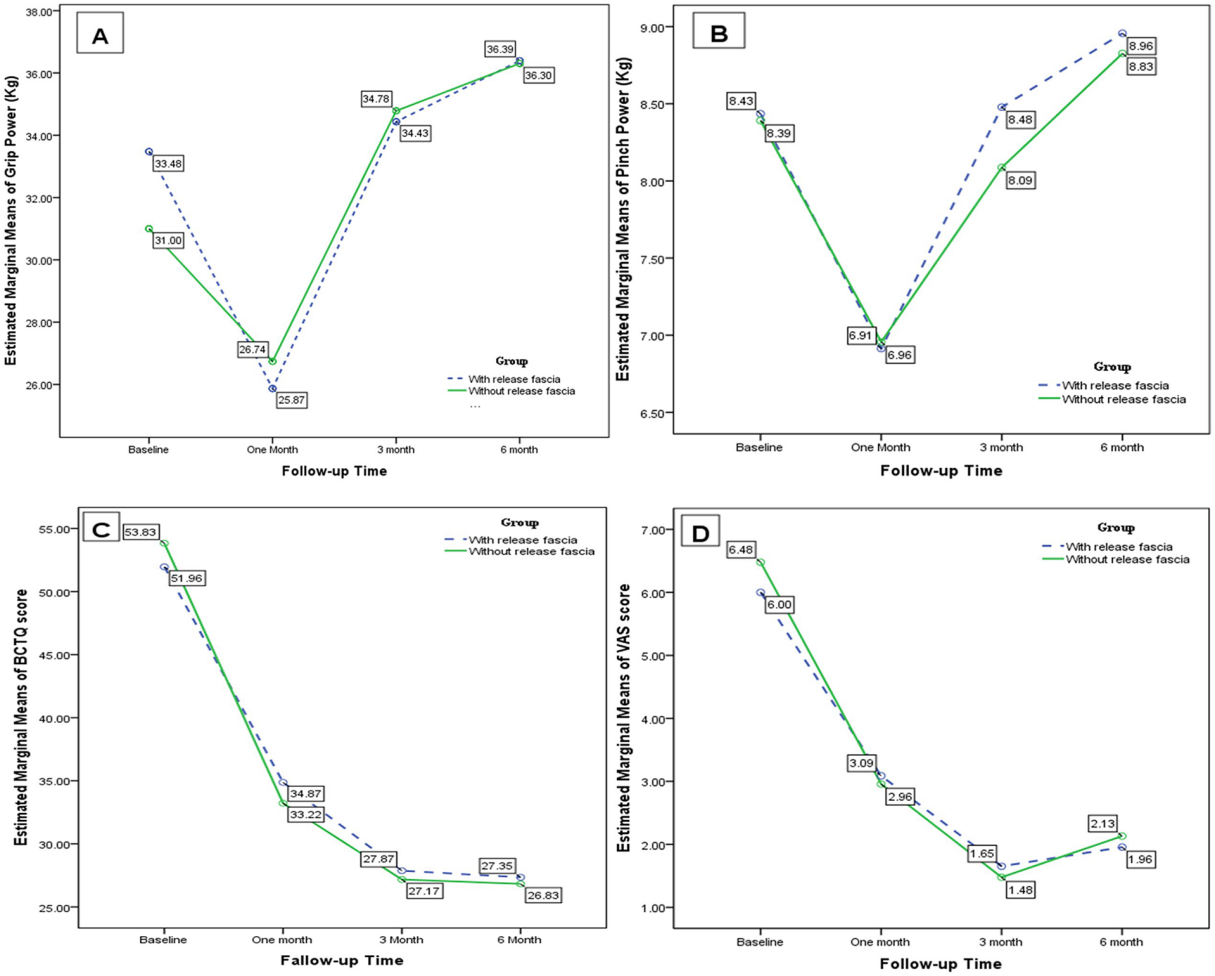
Changes in **(A)** grip strength, **(B)** pinch strength, **(C)** BCTQ scores, and **(D)** VAS scores during the study course.

In both open surgery groups with and without the release of the antebrachial fascia, grip strength was reduced in the first month after surgery. However, general linear model analysis showed that it significantly improved in both groups at the end of the study [*F* (2.48–54.75) = 16.93; *P* < 0.001, *F* (2.65–58.45) = 14.84; *P* < 0.001, respectively]. However, there was no statistically significant difference in grip strength variability between the two groups at any time in the study [*F* (2.68, 118.23) = 0.88; *P* = 0.443] (Table 1, Figure 3A).

**Table 1:** Comparison of clinical outcomes between the two groups.

Clinical outcome	With the release of the antebrachial fascia	Without the release of the antebrachial fascia	*P*-value
Grip 0 (before surgery)	33.47 ± 12.47	31.00 ± 11.17	0.482
Grip 1 (1 month after surgery)	25.86 ± 7.48	26.73 ± 7.92	0.704
Grip 3 (3 months after surgery)	34.43 ± 11.58	34.78 ± 10.38	0.915
Grip 6 (6 months after surgery)	36.39 ± 11.57	36.30 ± 9.79	0.978
Pinch 0 (before surgery)	8.43 ± 2.59	8.39 ± 2.69	0.956
Pinch 1 (1 month after surgery)	6.91 ± 2.23	6.95 ± 2.24	0.948
Pinch 3 (3 months after surgery)	8.47 ± 2.69	8.08 ± 2.69	0.625
Pinch 6 (6 months after surgery)	8.95 ± 2.20	8.82 ± 2.18	0.841
BCTQ 0 (before surgery)	51.95 ± 15.22	53.82 ± 15.81	0.685
BCTQ 1 (1 month after surgery)	34.86 ± 13.58	33.21 ± 10.60	0.648
BCTQ 3 (3 months after surgery)	27.86 ± 8.69	27.17 ± 8.07	0.780
BCTQ 6 (6 months after surgery)	27.34 ± 10.75	26.82 ± 11.58	0.875
VAS 0 (before surgery)	6.00 ± 2.62	6.47 ± 2.60	0.539
VAS 1 (1 month after surgery)	3.08 ± 2.96	2.95 ± 2.70	0.877
VAS 3 (3 months after surgery)	1.65 ± 2.40 (SE = 0.50)	1.47 ± 1.87 (SE = 0.39)	0.786
VAS 6 (6 months after surgery)	1.95 ± 2.38 (SE = 0.49)	2.13 ± 2.45 (SE = 0.51)	0.809
Satisfaction (1 month after surgery)	7.47 ± 2.44	7.86 ± 2.11	0.565
Satisfaction (3 months after surgery)	8.47 ± 1.50	8.69 ± 1.39	0.614
Satisfaction (6 months after surgery)	8.39 ± 2.03	8.26 ± 2.13	0.833

BCTQ, Boston Carpal Tunnel Syndrome Questionnaire; VAS, visual analog scale; SE, standard error.

Pinch strength decreased after the first month after surgery, but it finally improved in both groups with and without the release of the antebrachial fascia [*F* (2.56–56.47) = 5.80; *P* = 0.003, *F* (2.64–58.15) = 7.23; *P* = 0.001, respectively]. At the baseline and first, third, and sixth months after the surgery, there was no significant difference in pinch strength between the two groups [*F* (2.64, 116.25) = 0.158; *P* = 0.905] (Table 1, Figure 3B).

**Figure 4:**
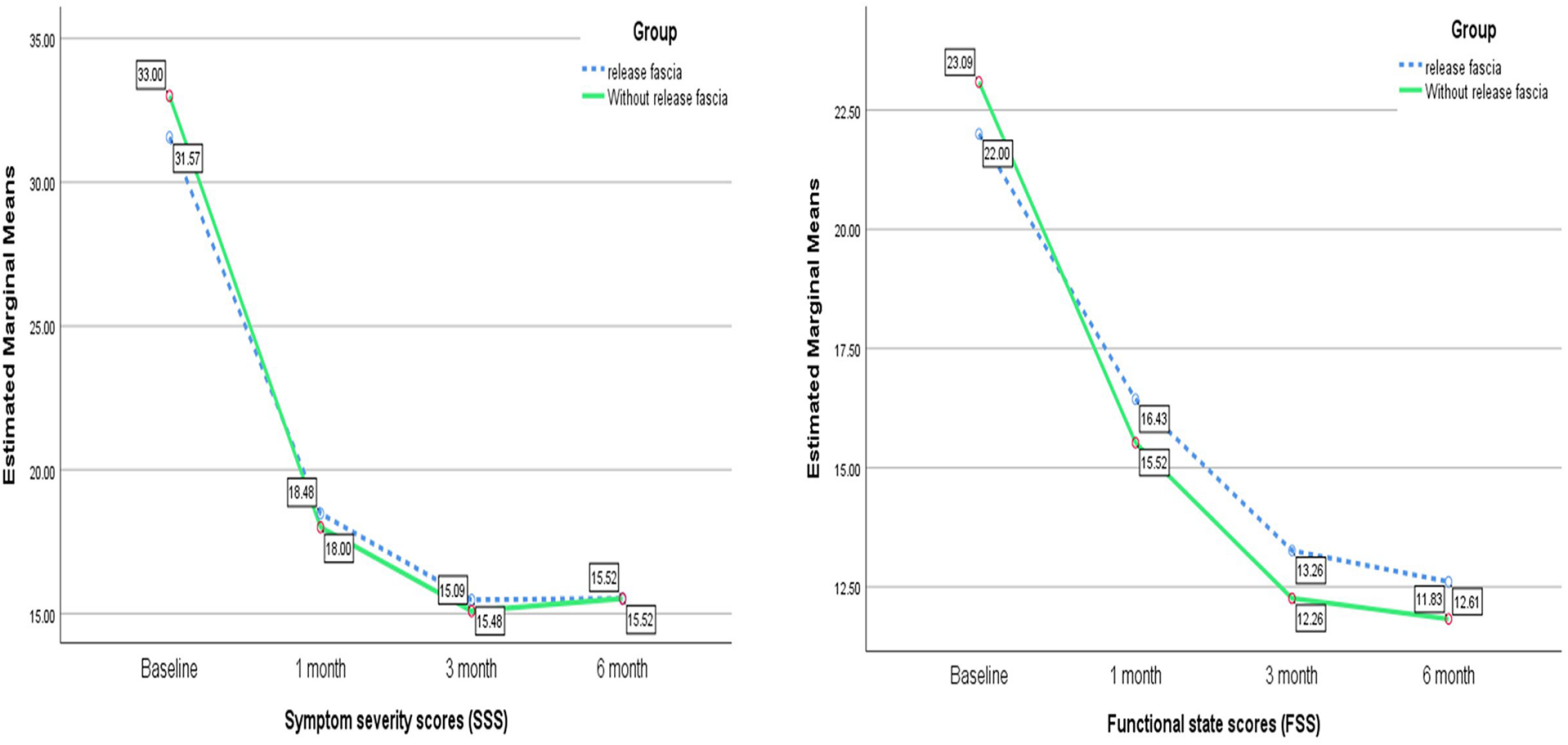
Changes in BCTQ subgroup scores (SSS and FSS) during the study course.

Open carpal tunnel surgery with and without the release of the antebrachial fascia significantly improved the BCTQ score [*F* (2.20–48.48) = 30.05; *P* < 0.001, *F* (2.06–45.33) = 34.30; *P* < 0.001, respectively]. However, there was no significant difference in the BCTQ score between the two groups [*F* (2.15, 94.66) = 0.246; *P* = 0.246] (Table 1, Figure 3C). In the subgroup analysis of the BCTQ, the mean scores of SSS and FSS were found to be significantly decreased in each group at the end of the study (*P* < 0.001); however, no significant difference was observed between the two groups (*P* > 0.05) (Table 2, Figure 4).

**Table 2:** Comparison of BCTQ subgroup scores between the two groups.

BCTQ subgroup score	With the release of the antebrachial fascia	Without the release of the antebrachial fascia	*P*-value
SSS 0 (before surgery)	31.56 ± 8.27	33.00 ± 8.85	0.573
SSS 1 (1 month after surgery)	18.47 ± 7.14	18.00 ± 6.55	0.814
SSS 3 (3 months after surgery)	15.47 ± 5.61	15.52 ± 6.51	0.768
SSS 6 (6 months after surgery)	15.52 ± 5.61	15.52 ± 6.51	1
FSS 0 (before surgery)	22.00 ± 6.69	23.08 ± 7.15	0.597
FSS 1 (1 month after surgery)	16.43 ± 6.78	15.52 ± 4.50	0.593
FSS 3 (3 months after surgery)	13.26 ± 4.92	12.26 ± 4.57	0.480
FSS 6 (6 months after surgery)	12.60 ± 5.73	11.82 ± 5.399	0.636

BCTQ, Boston Carpal Tunnel Syndrome Questionnaire; SSS, symptom severity scores; FSS, functional state scores.

Pain severity significantly improved in patients with and without the release of the antebrachial fascia [*F* (2.58–56.86) = 16.53; *P* < 0.001, *F* (2.01–44.37) = 26.61; *P* < 0.001, respectively]. Nevertheless, there was no statistically significant difference between the two groups [*F* (2.40, 105.69) = 0.217; *P* = 0.843] (Table 1, Figure 3D).

Furthermore, a multivariate analysis using logistic regression was performed to assess the possible effect of demographic variables on the measured factors (i.e., SSS, VAS, FSS, BCTQ, grip, pinch, and satisfaction). There was no significant relationship between the incidence of the mentioned measured factors and demographic variables.

### Secondary outcomes

**Figure 5:**
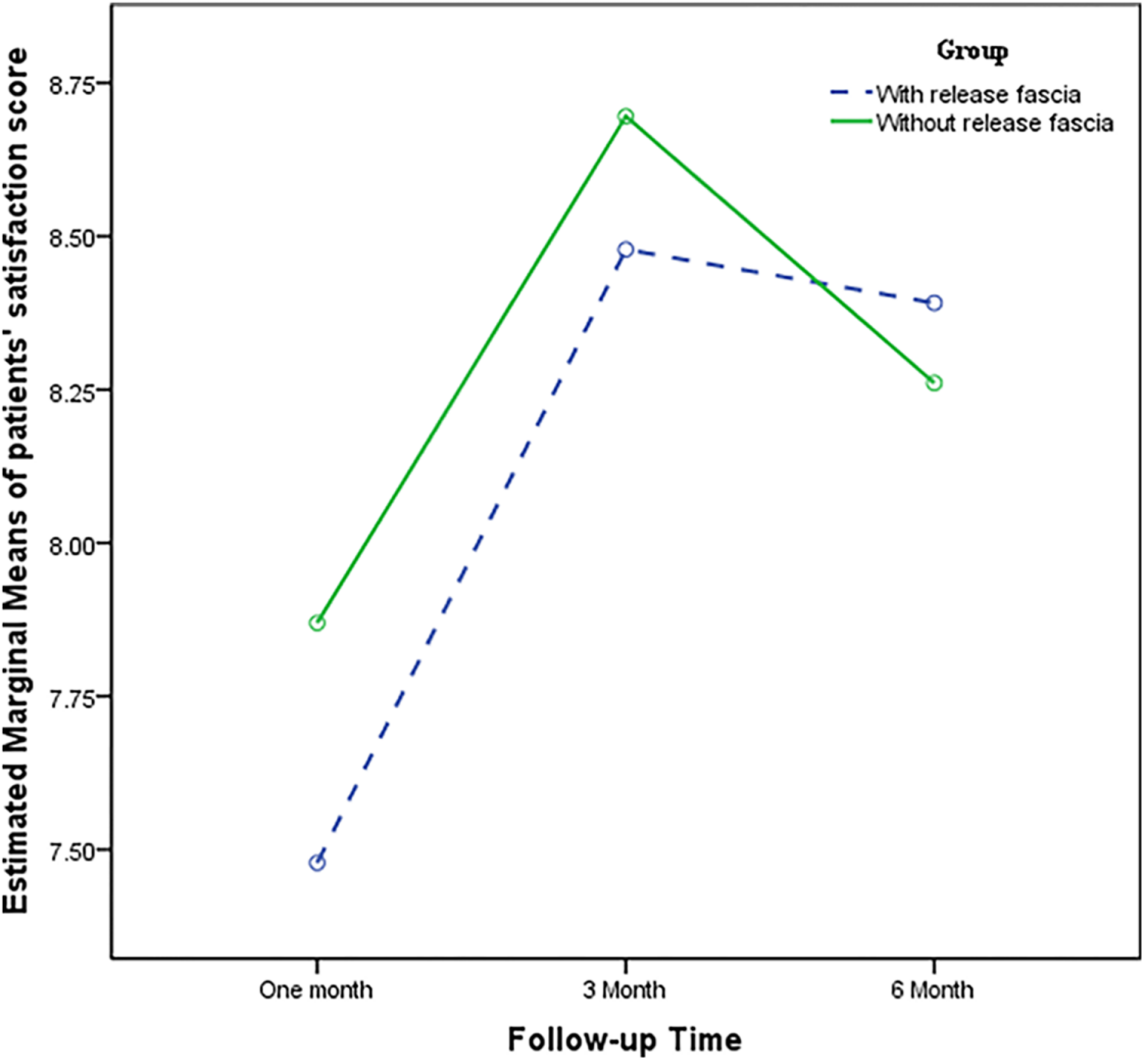
Comparison of patient satisfaction score.

Although the paired *t*-test showed a significant improvement in patient satisfaction at 3 months after open surgery with the release of the antebrachial fascia (*P* = 0.045), the general linear model test did not confirm this finding [*F* (1.59–35.16) = 2.91, *P* = 0.078]. Patient satisfaction in the open surgery group without the release of the antebrachial fascia did not show a statistically significant difference during the follow-up [*F* (1.98–43.72) = 1.48, *P* = 0.237]. Furthermore, patient satisfaction was not significantly different between the two groups [*F* (1.88–82.94) = 0.321, *P* = 0.714] (Table 1, Figure 5).

## Discussion

Carpal tunnel syndrome is a common neuropathy with a high annual incidence, primarily affecting middle-aged individuals and reducing their quality of life and ability to perform daily activities. Symptoms like numbness, pain, and weakness in grip and pinch strength are the main problems faced by patients. It has been shown that the fibrous layers of the fascia are responsible for creating increased pressure on the median nerve in the carpal tunnel.

A minimally open carpal tunnel release procedure involves making a small incision, typically about 1–2 cm, in the palm to access and cut the transverse carpal ligament. This approach aims to minimize scarring, reduce recovery time, and decrease postoperative discomfort.

In contrast, the extended open carpal tunnel release procedure involves a larger incision, usually extending from the palm to the wrist, to provide a more comprehensive view of the carpal tunnel. This allows the surgeon to ensure complete release of the transverse carpal ligament and address any additional anatomical issues. However, it generally results in a longer recovery period, more scarring, and potentially more postoperative pain compared to the minimally open technique. Endoscopic carpal tunnel release involves one or two small incisions, one in the wrist and possibly one in the palm. An endoscope, which is a thin, flexible tube with a camera, is inserted through the incision(s) to visualize the inside of the carpal tunnel. Guided by the endoscope, the surgeon cuts the transverse carpal ligament with a small blade or other cutting tool. The incisions are then closed with stitches or surgical tape. This technique has the advantage of smaller incisions, which can lead to less postoperative pain, a faster recovery period, and reduced scarring. However, it requires specialized equipment and training, and there is a slightly higher risk of damaging nearby structures due to limited visibility. In this study, our assumption is based on the notion that, anatomically, the antebrachial fascia does not exert pressure on the nerve and is not considered a compressive fibrous layer.

In the treatment of CTS, initial treatment is usually conservative, involving the use of NSAIDs, activity modification, and sometimes bracing (26). Several surgical techniques are available to reduce pressure on the median nerve, and these are used in CTS treatment and can be performed by open or endoscopic methods. These techniques have their advantages and disadvantages, but they are not the focus of this article. Traditionally, in open surgical techniques, deep and middle layers of the antebrachial fascia have been released for nerve decompression. Still, some studies suggested that the middle layer plays a proprioceptive role and recommended not injuring this part during surgery. In this study, we aimed to answer these questions: (1) Will patients achieve good outcomes in surgery without releasing the antebrachial fascia of the forearm? (2) Are there any differences in patient outcomes between surgeries that involve releasing the antebrachial fascia and those that do not? Oropeza-Duarte et al. compared mini-transverse and traditional reduced incisions in a case series and subsequently concluded in a prospective cohort study that the mini-transverse approach was more effective. However, the authors did not clarify whether the antebrachial fascia was addressed in both surgical techniques (27). In the studies by Ma et al. and Chen et al., two surgical methods were compared: the conventional technique and the mini-transverse incision and endoscopic technique, respectively. However, in both studies, the release of the antebrachial fascia was part of the surgical procedure (28, 29).

Nikkhah et al. published a technical tip suggesting the release of the proximal volar forearm fascia in carpal tunnel surgery to achieve better outcomes (30). Means et al. conducted a cadaveric study and concluded that releasing only the transverse carpal ligament, without incising the forearm antebrachial fascia, may lead to persistent high pressure on the median nerve (31). These results contradict ours, where we found no significant difference between patients with the antebrachial fascia released and those without it. When comparing these studies, it is notable that we conducted a clinical trial and used patient-reported questionnaires to evaluate the postoperative outcome.

Finally, in both open surgery groups with and without the antebrachial fascia release, grip and pinch strength, BCTQ scores, and pain severity significantly improved at the end of the study, but there was no statistically significant difference between the two studied groups. In addition, patient satisfaction improved in both groups, but no significant difference was observed between them. Therefore, by avoiding the release of the antebrachial fascia, proprioception is protected and the risk of iatrogenic injury to the median nerve and its branches is reduced, whereas releasing the antebrachial fascia will provide no improvement in outcomes.

In the current study, we encountered some limitations. First, some patients had incomplete follow-up visits, so we had to exclude them from the study. In addition, there are several problems related to endoscopic carpal tunnel release, including (1) the procedure is technically demanding; (2) a limited visual field that prevents inspection of other structures; (3) the vulnerability of the median nerve, flexor tendons, and superficial palmar arterial arch; (4) the inability to easily control bleeding; and (5) the limitations imposed by mechanical failure. For future investigations, it will be beneficial to perform this modification in the endoscopic technique and compare it with the traditional method. In addition, the outcomes can be assessed in long-term follow-ups.

## Data Availability

The original contributions presented in the study are included in the article/Supplementary Material, further inquiries can be directed to the corresponding author.
